# Stabilizing the Anode and Cathode Interface Synchronously via Electrolyte-Triggered Hydrogel Interphase for Zinc Metal Batteries

**DOI:** 10.1007/s40820-025-02051-1

**Published:** 2026-01-13

**Authors:** Xinze Cai, Xin Li, Jiahui Liang, Jiazhen Qiu, Wenkuo Lin, Chunlong Dai, Zifeng Lin, Jiangqi Zhao

**Affiliations:** 1 College of Materials Science and Engineering, Sichuan University, Chengdu, 610065 People’s Republic of China; 2 State Key Laboratory of Polymer Materials Engineering, Polymer Research Institute at Sichuan University, Chengdu, 610065 People’s Republic of China

**Keywords:** Zinc metal batteries, Aqueous electrolyte, Metal anode interfacial engineering, Solid-electrolyte interphase

## Abstract

The advancement of aqueous zinc metal batteries (ZMBs) is constrained by intrinsic interfacial issues in aqueous electrolyte systems. Here, using numerical simulation, we decipher the multi-scale causes of interfacial instability, elucidating the synergistic effect of macroscopic ineffective regions and microscopic passivation. Based on the analysis, we develop an electrolyte-triggered interphase construction strategy to resolve the interfacial failure. This strategy couples the in situ formation of hydrogel interphase on both the anode and cathode with the electrolyte filling process, thereby (1) facilitating contact between electrodes and the separator; (2) promoting anode reversibility through inducing a bilayer SEI that enhances Zn^2+^ desolvation kinetics and blocks electron tunneling; (3) ensuring long-term cathode cycling stability via restricting the irreversible dissolution of MnO_2_ and side-reactions. The resultant Zn metal anode exhibited a near-unity Coulombic efficiency (99.5%) for Zn plating/stripping at an extremely low current density of 0.1 mA cm^−2^ and the Zn/MnO_2_ full cell sustained 2000 full-duty-cycles with an exceptionally low decay rate of 0.0051% per-cycle. This work unlocks an alternative angle for promoting practical ZMBs toward more sustainable energy storage systems.

**Supplementary Information:**

The online version contains supplementary material available at 10.1007/s40820-025-02051-1.

## Highlights

- Decipher the multi-scale causes of interfacial instability in aqueous electrolyte systems via numerical simulations.
- Develop an electrolyte-triggered interphase construction strategy to achieve synergistic regulation of both the anode and cathode.
- Achieve high Coulombic efficiency (99.5%) and long-term cycling stability (over 6000 h) at ultra-low current density (0.1 mA cm^−2^) in zinc metal batteries.

**Supplementary Information:**

The online version contains supplementary material available at 10.1007/s40820-025-02051-1.

## Graphical abstract

**Figure:**
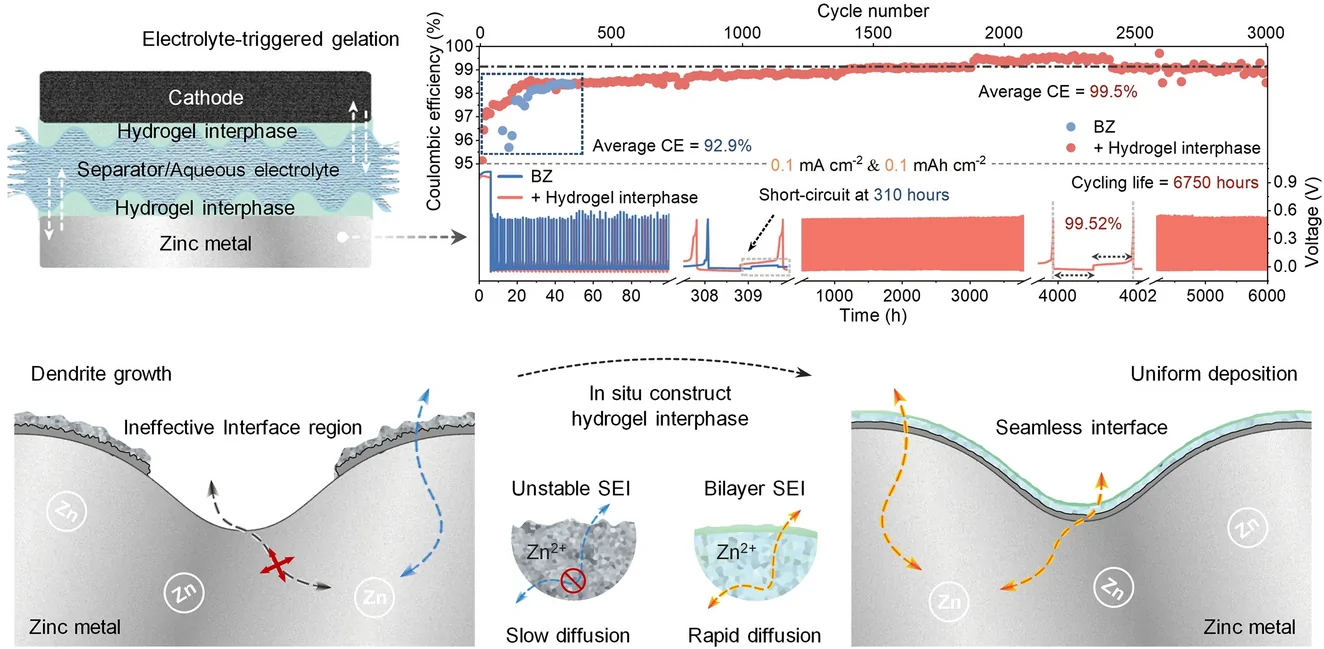

## Introduction

Aqueous zinc metal batteries (ZMBs) offer distinct advantages in terms of intrinsic safety, environmental friendliness and manufacturing cost, rendering them suitable to grid-scale energy storage systems [1, 2]. However, the natural properties of water cause a series of interfacial issues in such aqueous batteries, thus impeding the realization of this vision [3–5]. Specifically, (1) versus organic electrolyte, aqueous solvent is incapable of forming beneficial organic components of the solid-electrolyte interphase (SEI). Worse, the water-induced hydrogen evolution reactions (HER) will lead to a substantial accumulation of electrochemically inert alkali salt, forming a structurally loose and chemically unstable SEI that significantly reduces the coulombic efficiency (CE) of the battery [6]. (2) Anion-derived inorganic components of the SEI (such as ZnF_2_, ZnS, etc.) is susceptible to dissolution or transformation in aqueous solutions [7]. This, in turn, leads to further destabilization of the SEI in aqueous electrolyte, inducing severe interfacial electron tunneling and persistent electrolyte consumption. (3) The restricted contact area between the electrode and the separator, in conjunction with the constrained hydrophilicity of the electrode, collectively hinders the complete wetting of the electrode by the electrolyte, giving rise to ineffective interface regions on the electrode surface [8]. These regions engender the exacerbation of overall inhomogeneous distribution of ion fluxes at the electrode interface, leading to the formation of dendritic Zn and result in rapid short-circuit failure of ZMBs [9].

Interphase regulation offers a potential solution to the aforementioned problems. Ideally, at the macroscopic level, the interphase should exhibit both good electrode affinity and ductility to compensate for the uneven solid–solid contact between the electrode and the separator, thereby homogenizing the distribution of ion concentration and electric field at the electrode interface [10]. At the microscopic level, the interphase should exhibit complete electronic isolation, favorable ionic conductivity and sufficient mechanical toughness and resilience to ensure the long-term cycling stability of ZMBs [11]. Unfortunately, achieving the idealized interphase in the aqueous electrolyte system is still elusive. Within existing strategies, although electrolyte engineering can induce the formation of organic–inorganic hybrid SEI through functional electrolyte additive, it remains challenging to achieve high-performance SEI design and modulate macroscopic interfaces [12–14]. On the other hand, while artificial interface layer permits a high degree of customization of interfacial properties, this methodology carries potential risks of interface layer rupture and dislodgement during cycling, and faces challenges in achieving close contact between the interface layer and the electrode/separator [15, 16]. Therefore, to enable the coexistence of multi-scale functionality and stability, it is crucial to explore alternative platforms beyond conventional interphase regulation engineering.

**Fig. 1:**
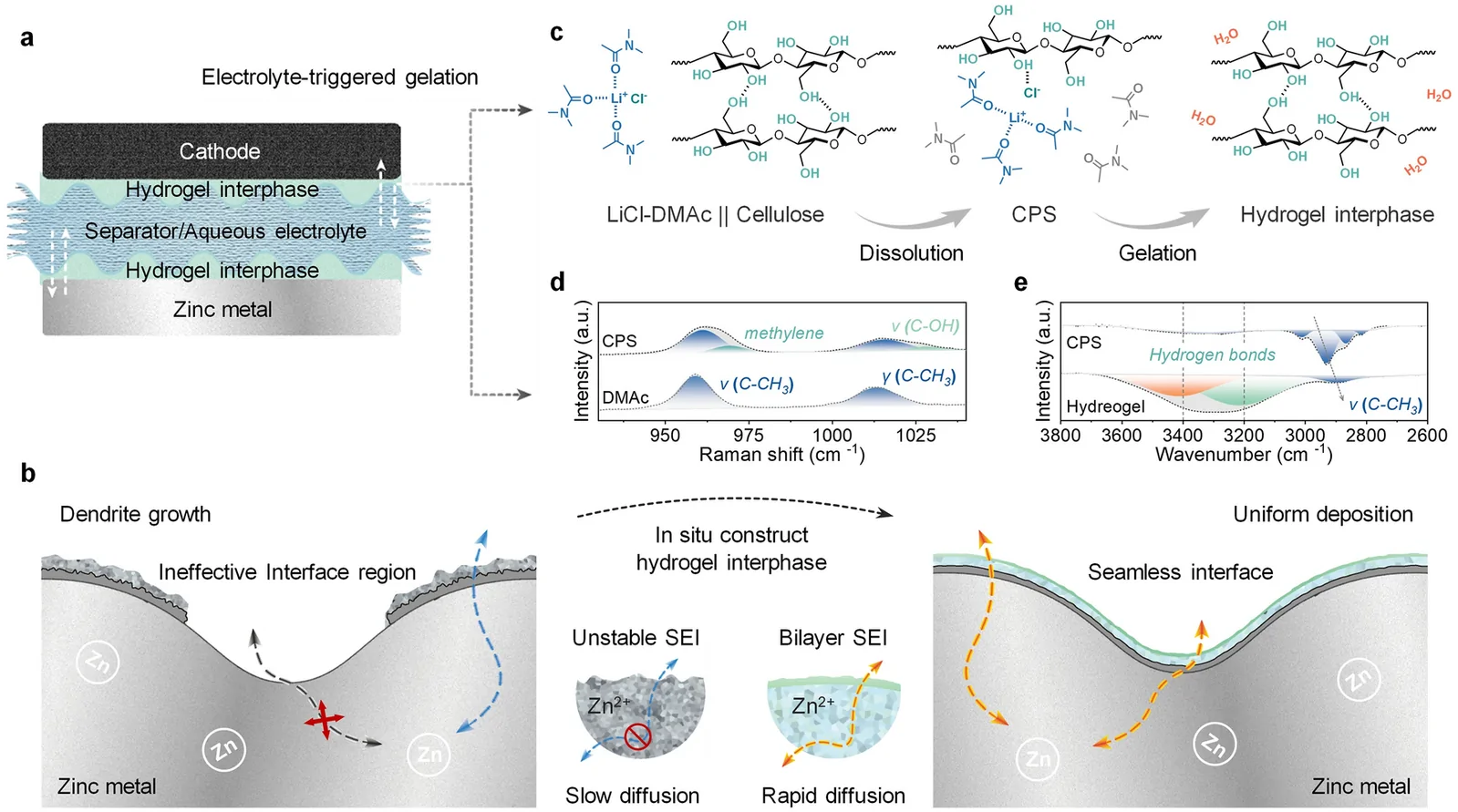
The design principle of the electrolyte-triggered interphase construction strategy. **a** Schematic of the electrolyte-triggered interphase construction strategy. **b** Conventional aqueous batteries suffer from interfacial mismatch and the absence of a stable SEI, resulting in the limited ion migration. The strategy facilitates the formation of seamless interface and bilayer SEI with high ion diffusion efficiency. **c** Schematic illustration of the hydrogel interphase formation process. **d** Raman spectra of DMAc and GPS and **e** FT-IR spectra of GPS and the hydrogel interphase

Here, inspired by the solvent-exchange approach in gelation chemistry, whereby replacing a good solvent (one that readily dissolving the target polymer) with a poor solvent (one that poorly dissolving the target polymer) to disturb the solvent-polymer interactions and form the tough gel network [17], we develop an electrolyte-triggered interphase construction strategy. This strategy, through pre-coating the electrode surface with polymer solution and exploiting the solvent exchange between water and the initial good solvent to drive gelation, synchronizes the electrolyte filling process with the in situ formation of hydrogel interphase on both the anode and the cathode (Fig. 1a). Exploiting the highly fluid of the polymer solution prior to gelation, this hydrogel interphase exhibits high continuity, eliminating macroscopic ineffective interface regions. Meanwhile, through electrochemical decomposition of the polymer solution at the interface, this hydrogel interphase induces the formation of a bilayer SEI to supersede unstable passivation layer in the conventional aqueous electrolyte system. The inner layer of the bilayer SEI, constituting of anion-derived inorganic species, is capable for enhancing the Zn^2+^ desolvation efficiency. The outer layer, consisting of polymer main-chained and the good-solvents-derived organic species contributes to the electronic isolation efficiency and chemical–mechanical stability of the bilayer SEI (Fig. 1b). With these merits, Zn/Cu half cells with hydrogel interphase achieve consistently stable cycling to operate over 6,000 h, with a high average CE of 99.5% at extremely low current density (0.1 mA cm^−2^). Moreover, this hydrogel interphase on cathode significantly suppresses the side-reaction and the transition metals dissolution, enabling Zn/MnO_2_ full cells exhibit an exceptional capacity retention rate of over 90% even after 2,000 full-duty-cycles, and the assembled Zn/MnO_2_ pouch cells deliver exceptional long-term cycling ability, even with a low negative/positive electrode capacity ratio (N/P ratio) of 1.42.

## Experimental Section

### Materials

Medical-grade absorbent cotton was purchased from Dalian Yangrun Trading Co., Ltd., China. Anhydrous Lithium chloride (LiCl), Dimethylacetamide (DMAc), methanol (MT), zinc sulfate heptahydrate (ZnSO_4_·7H_2_O), potassium permanganate (KMnO_4_), manganese sulfate (MnSO_4_) and N-methyl-2-pyrrolidone (NMP) were purchased from Sigma-Aldrich, China.

### Preparation of the Hydrogel Interphase

This experiment employs medical-grade absorbent cotton, primarily composed of cellulose (99%) and containing negligible amounts of other constituents such as lignin or hemicellulose, as the polymeric raw material. Firstly, 40 g of medical-grade absorbent cotton was stirred in 400 mL of deionized water for 12 h. Following this, the solvent was squeezed out and the mixture was transferred to 400 mL of methanol, where it was stirred for a further 12 h. Then, the solvent was once more squeezed out and the mixture was transferred to 400 mL of a DMAc solution, where it was stirred for a further 12 h. The activated cotton was then obtained after dried at 60 °C in a vacuum oven. At 60 °C, 8 g of LiCl was dissolved into 92 g of DMAc to obtain a LiCl-DMAc solution, after which 1 g of activated cotton was added and stirred continuously for 12 h to obtain a homogeneous cellulose precursor solution (CPS). The uniform CPS coating on the electrode surface was achieved through spin-coating, utilizing process parameters of 1,000 rad s^−1^ for 5 s, followed by acceleration to 3,000 rad s^−1^ for 10 s. The treated electrodes were then employed directly in the battery assembly, where the CPS underwent spontaneous gelation to form the hydrogel interphase following the injection of the electrolyte.

### Preparation of MnO_2_ Cathode

3 mmol MnSO_4_ and 2 mL 0.5 M H_2_SO_4_ were added to 90 mL deionized water with magnetic stirring to obtain a clear solution. Then, 20 mL 0.1 M KMnO_4_ aqueous solution was slowly added to the above solution. The mixture was stirred for 2 h at room temperature and then transferred into a Teflon-lined autoclave and heated at 120 °C for 12 h. Ultimately, the obtained MnO_2_ powder was washed repeatedly with distilled water and dried at 60 °C in a vacuum oven.

The cathode slurry was fabricated by mixing synthesized MnO_2_ powder (70 wt%), Ketjenblack (20 wt%), polyvinylidene fluoride (PVDF, 10 wt%) and N-methyl-2-pyrrolidone (NMP). Then the slurry was uniformly coated onto carbon cloth and dried in a vacuum oven at 80 °C for 12 h to obtain the MnO_2_ cathode.

### Battery Assembly

Glass microfiber filters (Whatman, GF/D, 16 mm in diameter) were used as the separator. The experimental procedures were carried out using three types of CR2032 coin cells: (1) Zn/Zn symmetric cell: utilizing Zn electrodes (commercial zinc foil, thickness 0.03 mm, diameter 14 mm) functioning as both cathode and anode, filled with 80 μL of 2 M ZnSO_4_ electrolyte. (2) Zn/Cu half cell, utilizing Zn electrode (commercial zinc foil, thickness 0.03 mm, diameter 14 mm) functioning as the anode and Cu electrode (commercial copper foil, thickness 0.05 mm, diameter 14 mm) functioning as the cathode, filled with 80 μL of 2 M ZnSO_4_ electrolyte. (3) Zn/MnO_2_ full cell, utilizing Zn electrodes (commercial zinc foil, thickness 0.03 mm, diameter 14 mm) functioning as the anode, MnO_2_ functioning as the cathode (active material loading of approximately 1.5 mg cm^−2^, diameter 14 mm), filled with 80 μL of 2 M ZnSO_4_ + 0.1 M MnSO_4_ electrolyte, operating within a voltage range of 1.0 to 1.8 V.

Pouch cells used for the experimental were Zn/MnO_2_ full cells, with Zn electrodes as the anode (commercial zinc foil, thickness 0.01 mm, size 4 cm × 5 cm, weight 85 mg), MnO_2_ as the cathode (active material loading of approximately 8 mg cm^−2^, size 4 cm × 5 cm), with 3 mL 2 M ZnSO_4_ + 0.1 M MnSO_4_ electrolyte. The aluminum-plastic film was utilizing as the encapsulation material.

All electrochemical performance data of the batteries (long-cycle performance, coulombic efficiency, rate capability, etc.) are derived from testing three independently prepared parallel sample batteries.

### Materials Characterization

Fourier transform infrared spectroscopy (FTIR) spectra were measured by Nicolet 6700. Raman spectroscopy was measured by LabRam HR Evolution. UV–visible absorption spectra were measured by the UV–visible absorption spectrophotometer (UV-1800PC). The morphologies and the elemental distribution were characterized by a field emission scanning electron microscope (FESEM, FEI Nova NanoSEM 230, 10 kV) equipped with an energy dispersive spectrometer (EDS). The FIB-SEM was employed to obtain the cross-section samples on the Helios 5 UC, and the SEI layer was investigated using the high-resolution TEM (Talos F200X G2). To study the spatial distribution of different components, we analyzed the processed SEI layers by TOF–SIMS (ION-TOF TOF.SIMS5). TOF–SIMS spectra and ion images were collected using Bi^3+^ ion beam accelerated at 30 keV and Cs^+^ accelerated at 1 keV. X-ray photoelectron spectra (XPS) were obtained by ESCALAB 250 Xi X-ray photoelectron spectrometer (Thermo Fisher) and Ar^+^ sputtering was employed for the XPS depth profile analysis (ESXCALAB Xi +). Nanoindentation tests utilize Nano Indenter (KLA-G200). Atomic force microscope (AFM) testing employs both the Kelvin probe force microscopy (KPFM) mode and the conductive atomic force microscopy (C-AFM) mode. Phase analyses and the crystal structures of the samples were investigated by X-ray diffraction (XRD) (Rigaku Mini Flex 600 diffractometer, Cu Kα radiation, λ = 1.5418 Å) with a scan rate of 10° min^−1^.

### Electrochemical Measurements

Galvanostatic charge–discharge (GCD) measurements were performed on the NEWARE CT-4008 T cell test instrument. The cyclic voltammetry (CV), chronoamperometry (CA), electrochemical impedance spectroscopy (EIS) spectra and linear sweep voltammetry (LSV) curves were collected by an electrochemical workstation (CHI660E, China).

CA investigations were conducted at a potential amplitude of 200 mV and a pulse width of 200 s. EIS was tested over a frequency range of 0.01 Hz to 1 MHz with an amplitude of 10 mV. LSV testing employed a three-electrode system (Zn electrode as the reference electrode, Zn electrode as the working electrode, Pt electrode as the counter electrode) to investigate hydrogen evolution reactions (HER) at different anodes. Prior to the LSV test, nitrogen is passed into the 2 M ZnSO_4_ electrolyte for a duration of half an hour to remove dissolved oxygen from the electrolyte. The LSV was performed from the open circuit voltage (OCV) down to -1 V at a scanning rate of 5 mV s^−1^ for the HER test. CV curves of Zn/MnO_2_ full cells were measured at a scan rate of 0.1 mV s^−1^ within the voltage range of 1 to 1.8 V. All experiments were conducted within a constant-temperature chamber maintained at 25 °C.

The desolvation energy (${E}_{a}$) was determined by measuring the impedance variation under different temperatures (10 – 60 °C) and then fitting the data according to the Arrhenius Eq. (1):

1 $$\frac{1}{{{ }R_{ct} }} = Ae^{{ - \frac{{E_{a} }}{RT}}}$$

Where ${R}_{ct}$ is the charge transfer resistance, A is the pre-exponential factor, R is the universal gas constant (8.314 JK^−1^ mol^−1^) and T is the absolute temperature.^1^

For in situ EIS test, a current density of 1 mA cm^–2^ was applied to intermittently charge/discharge the Zn/Zn symmetric cell. When charging/discharging for 0.1 mAh cm^−2^ (equivalent to 6 min) at each step, the cell was shelved for 30 min to reach equilibrium. Then, EIS measurements were conducted at open circuit potential over a frequency range of 0.01 Hz to 1 MHz with an amplitude of 10 mV. This charge/discharge–shelve–EIS testing cycle was repeated until the cell reached a cut-off capacity of 1 mAh cm^−2^.

### DRT Analysis

Distribution relaxation times (DRT) from the EIS data were calculated by MatlabR2023b with a toolbox of DRT-TOOLS developed by the research group of Professor Francesco Ciucci. DRT-TOOLS is freely available from the following site: https://github.com/ciuccislab.

DRT impedance, ${Z}_{\mathrm{DRT}}\left(f\right)$, at a frequency f, can be expressed as:

2 $${Z}_{\mathrm{DRT}}\left(f\right)=i2\pi f{L}_{0}+{R}_{\infty }+{\int }_{-\infty }^{+\infty }\frac{\gamma \left(\mathrm{log}\tau \right)}{1+i2\pi f\tau }d\mathrm{log}\tau$$

where ${L}_{0}$, ${R}_{\infty }$, τ, and $\gamma \left(\mathrm{log}\tau \right)$ are an inductance, an ohmic resistance, a timescale, and the DRT, respectively. In turn, the total polarization resistance, ${R}_{\mathrm{pol}}$, was computed using the following integral:

3 $${R}_{\mathrm{pol}}={\int }_{-\infty }^{+\infty }\gamma \left(\mathrm{log}\tau \right)d\mathrm{log}\tau$$

### Numerical Simulation

Finite element analysis of anodic electrodeposition was constructed in COMSOL Multiphysics 6.2 software, with a particular focus on the ion diffusion and migration in the electrolyte, which were described by the Nernst–Planck equation:

4 $${J}_{{\mathrm{Z}}{\mathrm{n}}^{2+}}=-{D}_{{\mathrm{Z}}{\mathrm{n}}^{2+}}({\nabla }_{{c}_{0}}-\frac{zF{c}_{0}}{RT}\nabla \varphi )$$

where ${J}_{{\mathrm{Z}}{\mathrm{n}}^{2+}}$ is Zn^2+^ flux, ${D}_{{\mathrm{Z}}{\mathrm{n}}^{2+}}$ is the diffusion coefficient, ${c}_{0}$ is the Zn^2+^ original concentration, z is the transferred electron numbers, F is the Faraday’s constant, R is the ideal gas constant, T is the Kelvin temperature and φ is the electrolyte potential.

The Zn metal anode surface exhibits a certain surface roughness, which can be implemented in COMSOL Multiphysics 6.2 using Gaussian random and uniform random distribution functions.

The deformed mesh functionality of COMSOL Multiphysics 6.2 was employed to dynamically simulate the impact of varying electrode surfaces on ionic mass transfer during the electrodeposition of Zn. In this simulation, the electrodeposition efficiency was assumed to be 100%, and secondary reactions were not considered.

## Results and Discussion

### Strategy Validation and Numerical Simulation of Electrodes Interface

In this work, cellulose is chosen as the polymer backbone for the interphase, and it is dissolved in a dimethylacetamide (DMAc)-lithium chloride (LiCl) solution to form a uniform cellulose precursor solution (CPS). Following the injection of 2 M ZnSO_4_ electrolyte, the good solvent (i.e., DMAc) will undergo spontaneous solvent exchange with the poor solvent (i.e., H_2_O), thereby facilitating the reformation of the inter-cellulose hydrogen bonds and triggering the in situ formation of hydrogel interphase (Figs. 1c and S1). Through spectroscopic characterization, this process is confirmed. The Raman spectroscopy reveals that, compared to the methyl stretching vibrations (${\nu }_{{\mathrm{C}-\mathrm{CH}}_{3}}$) and out-of-plane bending vibrations (${\gamma }_{{\mathrm{C}-\mathrm{CH}}_{3}}$) of the DMAc-LiCl solution at 958 and 1012 cm^−1^, CPS exhibits additional methylene stretching vibrations and primary alcohol stretching vibrations (${\nu }_{\mathrm{C}-\mathrm{OH}}$), indicating the dissolution of cellulose in the DMAc-LiCl solution (Fig. 1d) [18]. The Fourier transform infrared spectroscopy (FTIR) demonstrates that, compared to CPS, the hydrogel exhibits new broad vibrational bands at 3400 and 3200 cm^−1^, indicating the formation of cellulose-cellulose and cellulose-water hydrogen bonds. Moreover, due to the decrease of DMAc content, the carbonyl stretching vibration (${\nu }_{{\mathrm{C}-\mathrm{CH}}_{3}}$) of the hydrogel at 2931 cm^−1^ weakens, suggesting that the solvent exchange between DMAc and H_2_O causes the spontaneous gelation of CPS (Fig. 1e) [19]. Such solvent exchange phenomenon is also supported by the Raman spectroscopy of the residual electrolyte after gelation (Fig. S2).

**Fig. 2:**
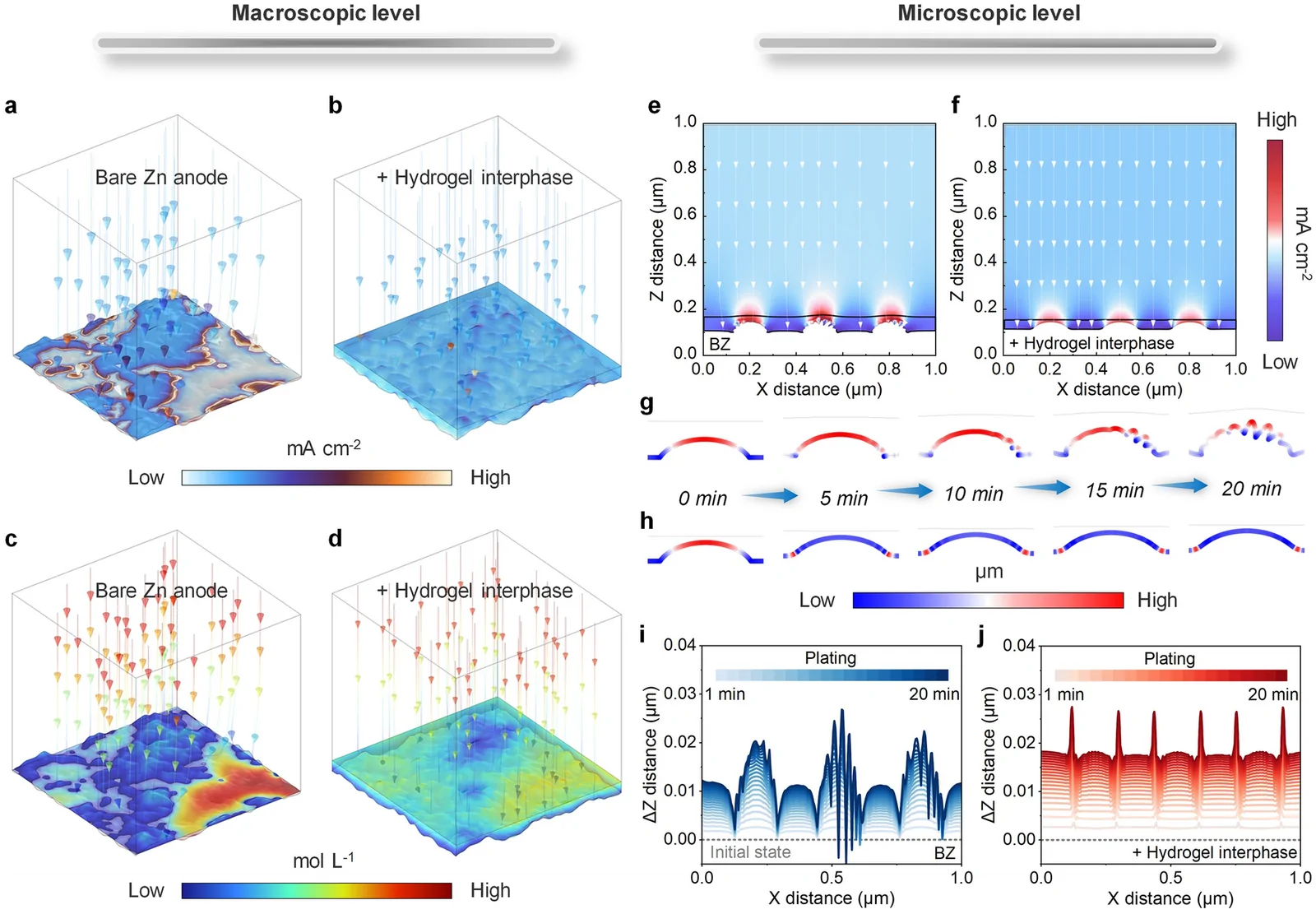
Numerical simulations of Zn plating process. **a** Local current density map of anodes without and **b** with hydrogel interphase, under the condition of an applied bias voltage of -0.5 V. **c** Zinc concentration map of anodes without and **d** with hydrogel interphase, under the condition of an applied bias voltage of -0.5 V. **e** Distribution of local current density on the BZ and **f** the hydrogel interphase modified anode along the direction of SEI thickness. **g** Evolution of anode surface morphology without and **h** with hydrogel interphase, when apply bias voltage of -0.5 V. **i** Height changes of anodes without and **j** with hydrogel interphase in the Z-axis direction during the plating process

To explain the causative factors of interfacial instability in aqueous electrolyte systems and validate the reliability of this strategy, the relationship between Zn^2+^ deposition behavior and anodic interface properties are analyzed through numerical simulation. Through combining the Gaussian random surface with the three-dimensional steady-state model, we compare the reaction distribution on different anode surfaces with an applied voltage (${E}_{app}$) of -0.5 V. As illustrated in Figs. 2a, c and S3, when the reaction reaches steady state, the existence of ineffective interfacial regions results in significant cation accumulation and localized high current densities on the bare Zn anode (BZ) surface. In contrast to the inhomogeneous ion transport at the interface of BZ, the incorporation of hydrogel interphase as a transition layer effectively equilibrates ion concentration and electric field distribution (Figs. 2b, d and S4). Such ion distribution behavior is effective in inhibiting the localized high current density or Zn^2+^ oversaturation induced dendrite formation, thereby confirming the positive impact of macroscopic interface contact regulation in achieving dendrite-free anodes [20].

The evolution process of the microscopic morphology of Zn anodes during deposition reactions was further investigated, by coupling the deformation geometry module with the electrochemical reaction module. Two different types of SEI were designed, one with a loose structure and lower ion diffusion efficiency (simulating the electrochemically inert alkali salt stacking layer formed by water decomposition), and the other with a dense structure and higher ion diffusion efficiency, to elucidate the effect of ion diffusion through the SEI on Zn^2+^ deposition [21]. The results of the simulation demonstrate that, when ion diffusion within the SEI is impeded, the minute protrusions on the anode surface tend to dendritic growth, accompanied by the emergence of conspicuous inhomogeneous electrical and Zn^2+^ concentration fields (Figs. 2e and S5). This particular growth pattern continuously generates finer new surface projections and exposes a larger reactive area, which will significantly diminish the thermodynamic stability and reversibility of the anode (Fig. 2g, i). In contrast, enhancing the ion diffusion efficiency within the SEI leads to the re-homogenization of the local chemical environment of the anode (Figs. 2f and S6), thereby reducing the discrepancy in deposition reaction rates across different locations on the anode (Fig. S7). Therefore, as the deposition time increases, the surface height at the junction between the anodic horizontal section and the protruding section increase significantly, ultimately forming a uniform and smooth electrode surface morphology (Fig. 2h, j).

### Effect of Hydrogel Interphase on Zn Plating/Stripping

**Fig. 3:**
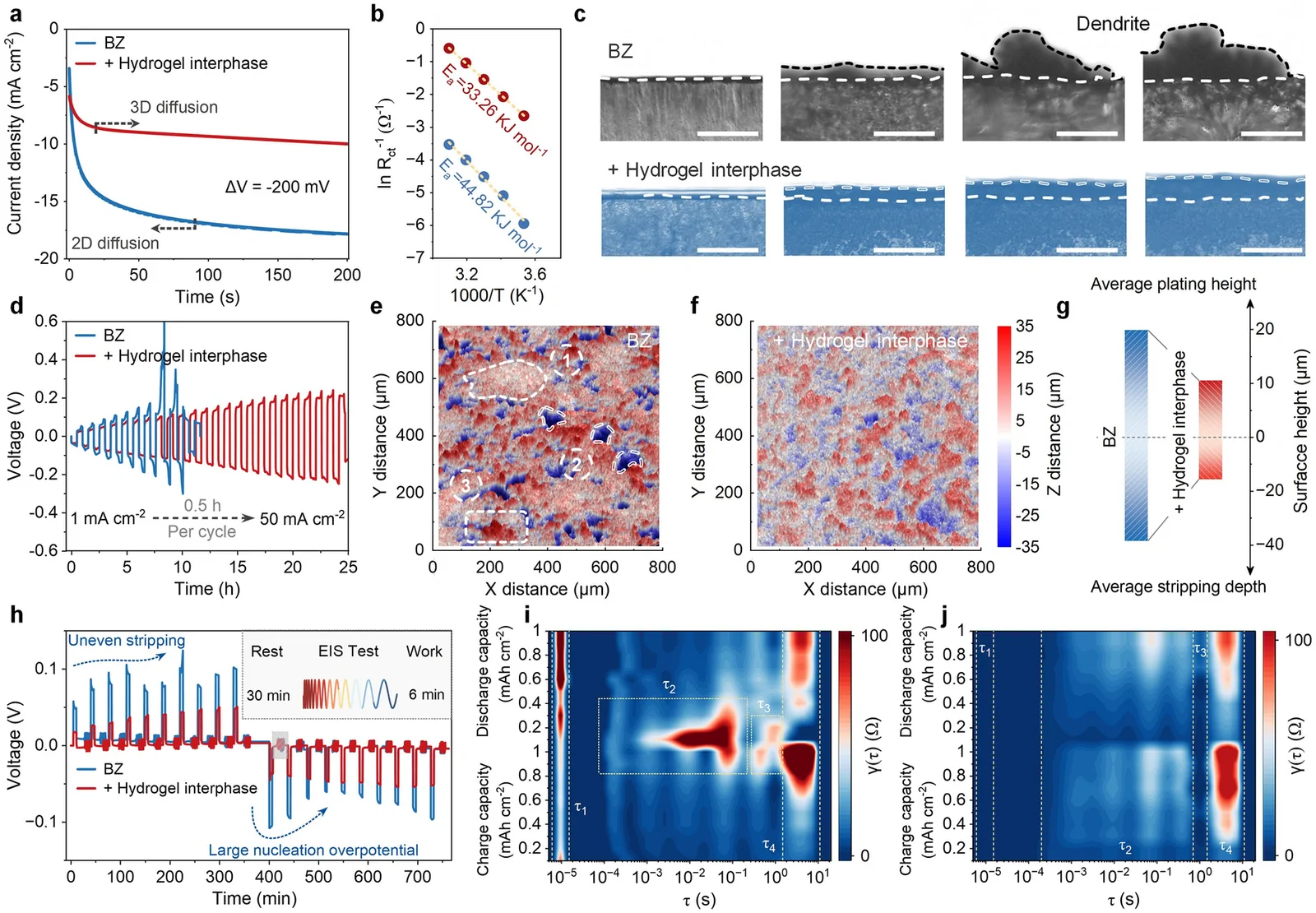
Plating/stripping behavior of Zn anode. **a** Chronoamperometry test of Zn/Zn symmetric cells with different anodes at a constant overpotential of 200 mV. **b** Arrhenius curves and the activation energy of the desolvation process. **c** in situ optical microscopy images of Zn plating (side view) at a current density of 10 mA cm^−2^ and up to 30 min. Scale bar, 200 µm. **d** Voltage–time profile of Zn/Zn symmetric cells at gradient current densities. Three-dimensional confocal laser microscopy images of **e** the BZ anode and **f** the hydrogel interphase modified anode after critical current density test. **g** Average surface height of different anodes. **h** Voltage–time profile of Zn/Zn symmetric cells during in situ EIS test. **i** Contour plots of corresponding DRT results of anodes without and **j** with hydrogel interphase

Based on the aforementioned-simulation results, the influence of the hydrogel interphase on Zn plating/stripping behavior was investigated. Chronoamperometry (CA) curves demonstrate that Zn^2+^ tends to stabilize three-dimensional (3D) diffusion on the anode with hydrogel interphase, while sustained Zn^2+^ two-dimensional diffusion is exhibited on the BZ anode, causing the continuously increased current density (absolute value) during the deposition process (Fig. 3a) [22]. As demonstrated in Figs. 3b and S8, the reduction in the desolvation energy (${E}_{a}$) of Zn^2+^ on the hydrogel interphase modified anode further indicates that the hydrogel interphase simultaneously enhances the kinetics of the deposition reaction while regulating Zn^2+^ diffusion. Such ionic characteristic facilitates the generation of more nucleation sites on the anode surface, thereby promoting homogeneous Zn^2+^ deposition [23]. Through utilizing optical microscopy, the morphological evolution of different anodes was visualized. As illustrated in Fig. 3c, as the deposition reaction progresses, discernible irregular protrusions are observed on the BZ while uniform electrodeposition can be consistently achieved on the anode with the hydrogel interphase. Furthermore, after 20 cycles, the BZ anode still exhibits substantial unreacted areas, while the hydrogel interphase modified anode displays a remarkably uniform reactive surface (Fig. S9). The regulation effect of the hydrogel interphase on Zn plating/stripping behavior enhances the maximum tolerable current of the Zn/Zn symmetric cell, resulting in stable cycling at extremely high current densities up to 50 mA cm^−2^ (Fig. 3d). Conversely, the BZ symmetric cell demonstrates elevated overpotential, resulting in cell short-circuiting at a current density of 16 mA cm^−2^. The SEM images of the Zn anode post critical current density test demonstrate that the cycled Zn anode surface with the hydrogel interphase is flat and smooth with no obvious dendrites, and the hexagonal Zn flake layer is in a dense horizontal stack (Fig. S10). In contrast, the BZ surface is covered with numerous dendritic deposits, which is in a loose vertical stack around the edges of stripping pits (Fig. S11). This phenomenon can be ascribed to the uneven-stripping-induced localized anode depletion, which exacerbates the asymmetry of the ensuing deposition process and promotes accelerated dendrite growth [24]. The confocal microscopy images reveal that the BZ exhibits various morphological features resulting from asymmetric plating/stripping, including localized unreacted areas induced by restricted surface contact (area 1); stripping pits (area 2); and dendrites (area 3) (Fig. 3e). In contrast, the Zn anode with the hydrogel interphase exhibits an overall identical homogeneous surface morphology (Fig. 3f), with reduced surface height undulations (Fig. 3g) and a lower average surface roughness (Fig. S12).

Notably, the experimentally measured results of anode morphology characteristics demonstrate a high degree of consistency with the simulation results. To elucidate the underlying causal relationship, in situ electrochemical impedance spectroscopy (EIS) was conducted at different stages of the charging/discharging process in the Zn/Zn symmetric cell (Figs. 3h and S13) (the experimental details are described in the Methods section), and the distribution of relaxation times (DRT) analysis was used to decouple the processes governing the EIS spectra [25]. As shown in Fig. S14, DRT plots of the EIS during the plating/stripping process exhibit four peaks within a timescale (τ) range of 10^–6^—10^1^ s, denoted as ${\tau }_{1}$—${\tau }_{4}$. During the initial charging process of the Zn/Zn symmetric cell with BZ, the peaks ${\tau }_{2}$, corresponding to Zn^2+^ diffusion through the SEI, remain essentially unchanged, which can be ascribed to the difficulty of forming stable SEI in the aqueous electrolyte system (Fig. 3i) [26]. This means that the stripping process will initiate at the energetically favorable location, continuing until the reserves of active Zn in this region are depleted before progressing to other areas, resulting in the periodic oscillations in the voltage response during the charging process (Fig. 3h) [27]. Subsequently, the intensity of peaks ${\tau }_{2}$ and ${\tau }_{3}$, which represents charge transfer process on the electrode, display significant increase in the final three cycles of the charging process and reach a maximum after the initial discharge, leading to the pronounced nucleation overpotentials during the discharge process. This indicates that the formative SEI on the BZ is characterized by ionic diffusion inefficiency and susceptibility to electrode reactions, rendering it incapable of effectively isolating solvated H_2_O and stabilizing electrode interfaces. Moreover, the intensity of peaks ${\tau }_{1}$ and ${\tau }_{4}$, representing the interfacial contact resistance and the diffusion of Zn^2+^ in the deposited layer, exhibited a marked increase as the discharging process progressed, which indicates the formation of a loose and non-uniform deposited layer on the surface of the BZ, leading to a sustained overpotential increase in the later stages of the discharge process [28]. In contrast, subsequent to the implementation of the hydrogel interphase, the intensity of peaks ${\tau }_{2}$ and ${\tau }_{4}$ gradually increases during the charge/discharge process, indicating that the hydrogel interphase can facilitate the formation of the stable SEI with elevated ionic diffusion efficiencies and the formation of compact deposited layers (Fig. 3j). While the intensity of peaks ${\tau }_{1}$ and ${\tau }_{3}$ maintains a consistent low level, confirming the effect of the hydrogel interphase in regulating macroscopic interface contact. The results further confirm the pivotal role of the hydrogel interphase in modulating the Zn plating/stripping behavior, thus highlighting the significant advantage of this strategy.

### Composition and Properties of SEI

**Fig. 4:**
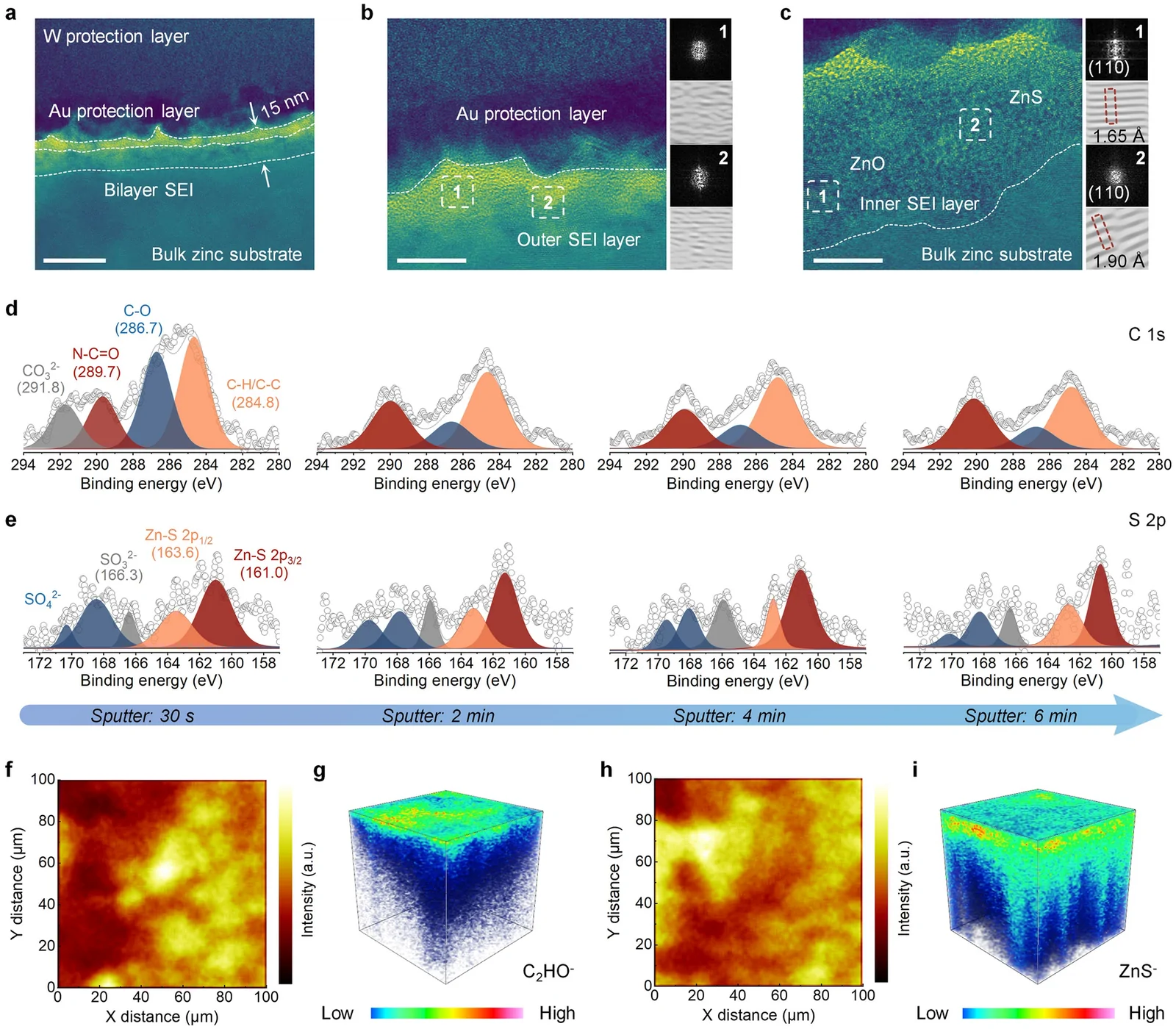
Structural and component characterizations of the hydrogel interphase-induced SEI. **a** SEM images of hydrogel interphase modified anodes cycled for 20 cycles. Scale bar, 20 nm. **b** and **c** HRTEM images, the fast Fourier transform images and corresponding inverse fast Fourier transform images collected at specific regions of hydrogel interphase modified anodes cycled for 20 cycles. Scale bar, 5 nm. XPS spectra with depth profiles of **d** C 1*s* and **e** S 2*p*. **f** TOF–SIMS 3D of C_2_HO^−^ and **g** corresponding 2D distributions from the hydrogel interphase-induced SEI. **h** TOF–SIMS 3D of ZnS^−^ and **i** corresponding 2D distributions from the hydrogel interphase induced SEI

The DRT plots indicate that the hydrogel interphase induces the formation of a stable SEI [29]. To understand the properties of the hydrogel interphase-induced SEI, the composition and structure of the cycled anode was investigated by observing the focused ion beam (FIB) prepared cross-section with transmission electron microscopy (TEM). The FIB cross-section of the hydrogel interphase modified anode displays a distinct SEI on the bulk zinc substrate, with a uniform thickness of approximately 15 nm and a dense bilayer structure (Fig. 4a). High-resolution transmission electron microscopy (HRTEM) and fast Fourier transform (FFT) mode reveals that the SEI consists of an amorphous outer layer (Fig. 4b) and an inner layer of inorganic components (Fig. 4c). Energy-dispersive X-ray spectroscopy elemental mappings (EDS) associated with the high-angle annular dark-field imaging (HAADF) delineate the spatial distribution of C, O, S, and Zn further confirmed the bilayer structure of the hydrogel interphase-induced SEI (Fig. S15).

Through X-ray photoelectron spectroscopy (XPS), the depth-profiled composition throughout the hydrogel interphase-induced SEI was quantitatively analyzed (Fig. S16). The C 1*s* spectra of the SEI can be deconvoluted into four components (Fig. 4d). The peaks at 284.8 and 286.7 eV are assigned to C–C/C–H and C–O bonds, respectively, the peak at 289.7 eV is assigned to the N–C=O bond, and the peak at 291.8 eV is ascribed to the carbonate. Where, the existence of the C–O bond can be attributed to the cellulose backbone, and the presence of N-containing groups can be attributed to the reduction of the good solvent (DMAc). After Ar^+^ sputtering for 2 min, the peak at 291.8 eV is fully eliminated, indicating the presence of carbonate signals is caused by contamination of the surface by CO_2_ in the atmosphere. Concurrently, the peak intensity of the C–O bond decreased significantly, while the peak intensity of the N–C=O bond remains relatively constant, which matches with the trend of the N 1*s* signals (Fig. S17), suggesting that organic components are predominantly concentrated in the outer layer of the SEI. The S 2*p* spectra indicates, along the depth direction of the SEI, the proportion of sulfur oxides decreases, while that of zinc sulfides (Zn–S) gradually increases (Fig. 4e). Furthermore, combined the Auger electron spectra with the Zn 2*p* spectra, the signals of ZnS and ZnO were identified, which signify that ZnS and ZnO collectively comprise the inorganic components within the inner layer of the SEI (Figs. S18 and S19). The spatial distribution of SEI components was analyzed by time-of-flight secondary ion mass spectrometry (TOF–SIMS). The signal intensity of C_2_HO^−^ fragments (cellulose-induced) demonstrate a rapid decrease along the perpendicular direction, which is consistent with the results of the XPS tests (Fig. 4f, g). Concurrently, the organic component fragments (CNO^−^, C^−^) are stacked atop the inorganic component (ZnS^−^, Zn_2_O^−^ and ZnSO^−^) (Figs. 4h, i and S20), further corroborating the bilayer structure of the SEI, with an outer organic layer and an inner inorganic layer.

TEM, XPS spectra and TOF–SIMS were also utilized to characterize cycled BZ (Figs. S21–S26). Overall, a porous, thick and inhomogeneous SEI appeared on the cycled BZ, consisting primarily of zinc sulfate hydroxide hydrate (6Zn(OH)_2_·ZnSO_4_·4H_2_O) (Fig. S27), a poor Zn^2+^ conductor, which result in retarded ion transport and elevated interfacial impedance, thereby significantly reducing the stability of the anode [30]. It is intriguing that Zn–S signals were also detected on the surface of the cycled BZ, verifying by TOF–SIMS, the presence of anion-derived inorganic components (ZnS) was confirmed (Figs. S28 and S29). However, this component is present only in negligible amounts within the SEI, rendering it incapable of providing effective interfacial protection. This phenomenon can be attributed to the inherent thermodynamic instability of the inorganic component within aqueous electrolyte systems. Following its formation, the inorganic component is susceptible to erosion by the aqueous solvent, resulting in its transformation [31]. This assertion is further substantiated by the apparently inhomogeneous surface distribution of SO_2_^−^ fragments in the cycled BZ (Fig. S30) and the high normalized intensity of Zn_2_SO_4_OH^−^ fragments (Fig. S31). These findings imply that the hydrogel interphase effectively stabilize the anion-derived inorganic SEI component, through the outer cellulose skeleton and the in situ formed organic SEI component, thereby constructing a stable bilayer SEI with high ionic diffusion efficiency in the aqueous electrolyte system.

**Fig. 5:**
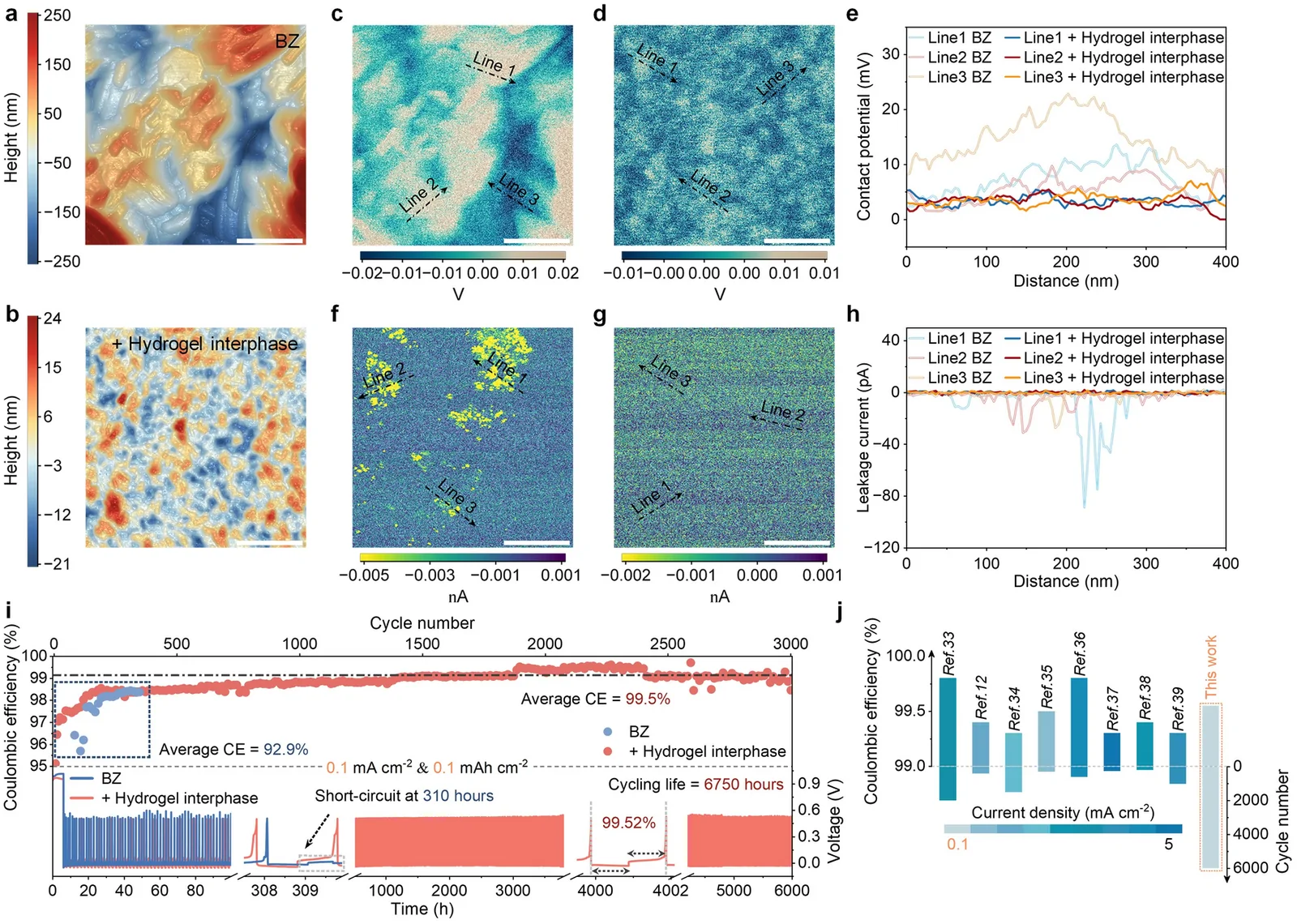
Physical characterization of the hydrogel interphase-induced SEI. **a** AFM images of topography of BZ anodes and **b** hydrogel interphase modified anodes after cycling. Scale bar, 1 µm. **c** Surface potential mapping of BZ anodes and **d** hydrogel interphase modified anodes after cycling. Scale bar, 1 µm.** e** Corresponding surface potential distributions for one-dimensional selected region. **f** Surface current mapping of BZ anodes and **g** hydrogel interphase modified anodes after cycling. Scale bar, 1 µm. **h** Corresponding surface current distributions for one-dimensional selected region. **i** The GCD potential profiles for the average CE measurement in Zn/Cu half cells at 0.1 mA cm^−2^ and 0.1  mAh cm^−2^. **j** Summary of published CE data of zinc anode

To conduct a more detailed investigation into the interphase physical properties, atomic force microscopy (AFM) was utilized (Fig. 5a, b). Combining two AFM operation modes, Kelvin probe force microscopy (KPFM) and conductive atomic force microscopy (C-AFM), the electronic isolation properties of different SEI were quantitatively assessed. The interfacial contact potential (${V}_{\mathrm{CP}}$) of the electrode with respect to the tip was determined via utilizing KPFM, and the electron work function of the electrode (${\varnothing }_{E}$) was further calculate by the following equation: ${\varnothing }_{E}={\varnothing }_{\mathrm{tip}}-{V}_{\mathrm{CP}}\bullet e$, where ${\varnothing }_{\mathrm{tip}}$ is the work function of the tip, and e is the electronic charge [32]. As shown in Fig. 5c, d, the ${V}_{\mathrm{CP}}$ of the hydrogel interphase induced SEI is considerably lower that of BZ. The one-dimensional selected region measurements of the surface potential further illustrate the higher electron work function of the hydrogel interphase induced SEI, which will significantly reduce the probability of electron escape (Fig. 5e). For electronic conductivity measurement (Fig. 5f, g), the hydrogel interphase-induced SEI has a notably lower surface leakage current than that of BZ (Fig. 5h). Moreover, the nanoindentation tests demonstrate that hydrogel interphase induced SEI exhibits a 2.2-fold increase in average Young's modulus (29.1 GPa) and a moderately decrease in nano-hardness (0.2 GPa) compared to the SEI on BZ (12.8/0.5 GPa) (Figs. S32 and S33). Therefore, unlike the formative SEI on BZ surface, which consists primarily of alkali slate, the bilayer SEI exhibit near-complete electronic isolation and excellent toughness, thereby allowing the hydrogel interphase modified anode to maintain exceptional interphasial stability throughout long-term cycling. The properties of the bilayer SEI significantly inhibit water-induced interfacial corrosion reactions (Fig. S34), enabling Zn/Cu half cells to cycle continuously and reliably for periods exceeding 6000 h while maintaining an extreme-high average CE of 99.5% at a remarkably low current density of 0.1 mA cm^−2^ (Fig. 5i). Compared to previously reported ZMBs, achieving near-unity CE at such a low current density is unprecedented (Fig. 5j), which further validates the efficacy of our strategy in modulating interphasial properties [12, 33–39].

### Electrochemical Performance of Full Cells

The interfacial issues at the cathode also hamper the development of ZMBs (in the case of MnO_2_, transition metals (TMs) dissolution and the accumulation of by-product significantly reduce the reversibility and the cycle stability of Zn/MnO_2_ cells) [40–42]. Benefit from the high applicability of this strategy, the hydrogel interphase was also constructed on the cathode surface to address the aforementioned-issues. The dissolved TMs in the electrolyte during cycling was traced by ultraviolet spectroscopy.

**Fig. 6:**
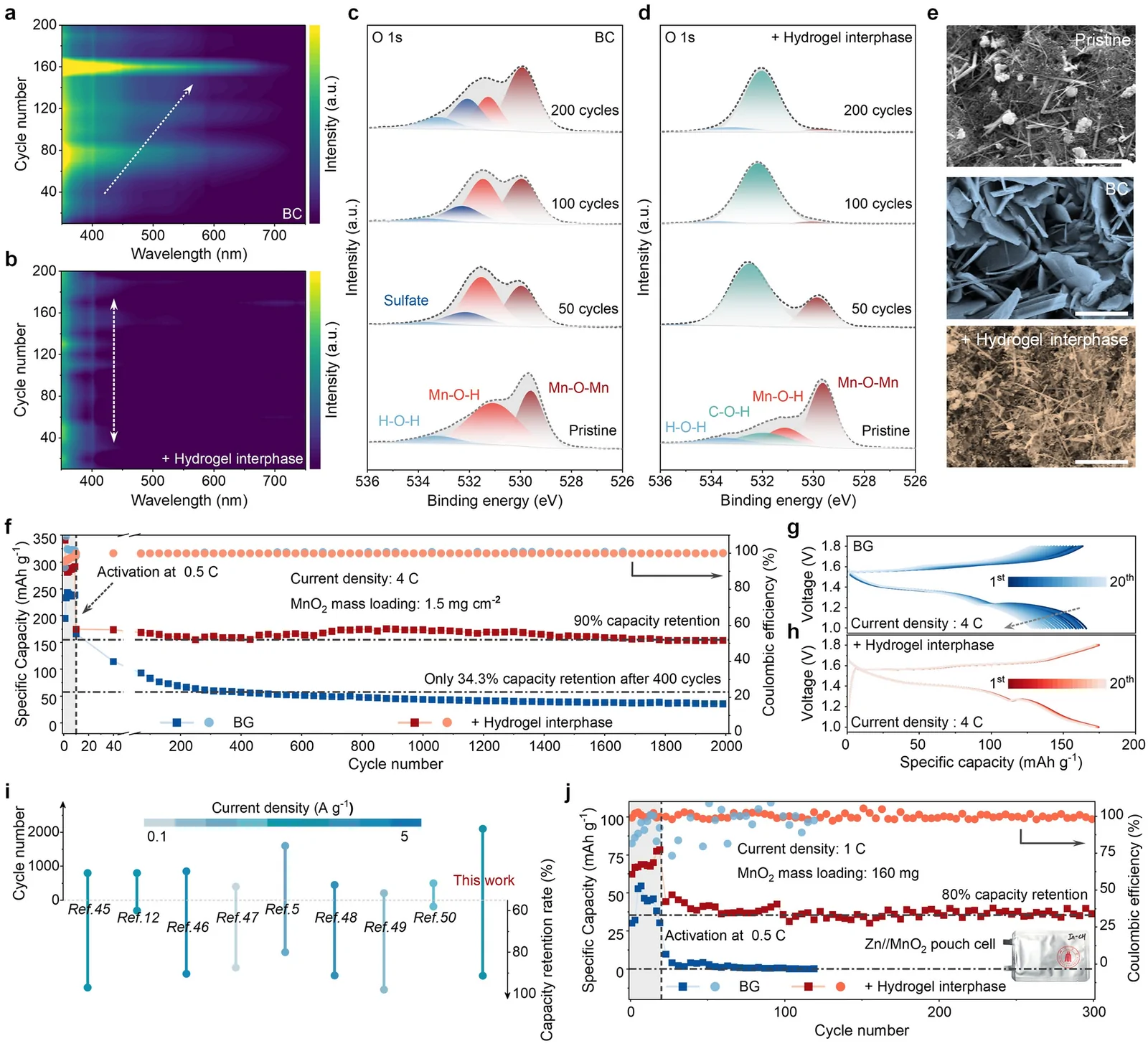
Electrochemical performance of Zn/MnO_2_ full cells. **a** Contour plots of ultraviolet–visible spectroscopy of Zn/MnO_2_ full cells without and **b** with hydrogel interphase. **c** XPS analysis for cathodes without and **d** with hydrogel interphase following cycling. **e** SEM images of different cathode. Scale bar, 200 µm. **f** Cycling performance of Zn/MnO_2_ full cells without (designated as blank control group (BG)) and with hydrogel interphase. **g** Voltage profiles of Zn/MnO_2_ full cells without and **h** with hydrogel interphase after different numbers of cycles. **i** Comparison of this work with state-of-the-art ZMBs. **j** Pouch cell cycling with N:P ratio of 1.42. Inset: digital image of the pouch cell

As illustrated in Fig. 6a, the ultraviolet absorption signals in the 400–600 nm band of the electrolyte of the Zn/MnO_2_ cells with bare cathode (BC) gradually increase upon cycling, which can be ascribed to the generation of intermediate Mn^3+^ and the formation of suspended MnO_2_ particles through the disproportionation reaction of Mn^3+^ [43]. By contrast, the electrolyte of the cell with the hydrogel interphase displays a substantial ultraviolet absorption signal exclusively within the 350–400 nm band, indicative of Mn^2+^ (Fig. 6b). Moreover, the XPS analysis was conducted on the cycled cathode to reveal the interfacial evolution. As shown in Fig. 6c, the sulfate component is detected on the cycled BC surface and the signal intensity of this component gradually increases with cycling. Conversely, the hydrogel interphase modified cathode exhibits a discernible organic component (C-O–H signal in the O 1*s* spectra), which progressively prevailed upon cycling and substantially impeded the formation of by-products, highlighting the consistent and stable interfacial protection effect of the hydrogel interphase (Fig. 6d). These XPS results agree with the SEM measurements, where a large amount of lamellar alkaline zincate adheres to the BC surface, while MnO_2_ particles assumed nanorod configuration can still be distinctly observed on the hydrogel interphase modified cathode (Figs. 6e, S35 and S36).

Enhanced interfacial stability enabled the Zn/MnO_2_ cell with the hydrogel interphase to demonstrate exceptional cycling performance, maintaining more than 2,000 cycles at a current density of 4C with an extremely low decay rate of 0.0051% per-cycle (1C = 308 mA g^−1^) (Figs. 6f and S37). More impressively, the second discharge platform of this Zn/MnO_2_ cell remained virtually unchanged during the first 20 cycles (Fig. 6g, h), highlighting the rapid construction of the hydrogel interphase at both cathode and anode, in addition to its effective interfacial stabilization [44]. This remarkably stable cycling marks a substantial advancement in the domain of ZMBs (Fig. 6i) [5, 12, 45–50]. Furthermore, Zn/MnO_2_ cells with the hydrogel interphase maintain 85.7%, 57.7%, 46.3%, and 38.8% initial capacity at discharging rates of 2C, 6C, 8C, and 10C, respectively (Fig. S38). Such enhanced interfacial kinetics were verified by cyclic voltammetry (CV) and EIS tests, where the cells with the hydrogel interphase consistently maintained reversible redox peaks and lower resistance upon cycling, revealing the durability and rapid interfacial kinetics of the hydrogel interphase (Figs. S39 and S40). With these merits, we further evaluated the electrochemical performance of single-layer Zn/MnO_2_ pouch cells with low N/P ratio (1.42), consisting of high-loading MnO_2_ cathode (8.0 mg cm^−2^) and ultrathin Zn anode (10 μm). Through ameliorating electrode interfacial environment, the pouch cell with the hydrogel interphase exhibits a capacity density of approximately 50 mAh g^−1^ (based on the mass of both electrodes) and delivers a stable cycling performance over 300 cycles at a current density of 1C, whilst the pristine pouch cell is almost completely inoperable (Fig. 6j).

## Conclusions

In conclusion, by employing the solvent exchange gelation, a novel electrolyte-triggered interphase construction strategy is developed to resolve the intrinsic interfacial issues in aqueous electrolyte system. The in situ formed hydrogel interphase eliminates inactive interface regions and establishes an anion/solvent-derived bilayer SEI with excellent chemo-mechanical stability and accelerated interfacial kinetics. Concurrently, this hydrogel interphase prevents the cathode deactivation, which is precipitated by the dissolution of TMs and the accumulation of alkaline zincate by-products, promising excellent cycling stability and outstanding performance for ZMBs even under practical conditions (high mass loading and low N/P ratio). Through elucidating the causative link between the interfacial properties and electrode reversibility from a multi-scale perspective, this work underscores the pivotal function of modulating interfacial characteristics in surmounting the interfacial challenges. Attributing to the substantial material design space of polymer chains and initial solvents, this strategy is foreseeable to illuminate the design of advanced interphase, thereby facilitating the development of practical ZMBs and contributing to the sustainability of the global energy.

## Supplementary Information

Below is the link to the electronic supplementary material.

Supplementary file1 (DOCX 20679 kb)
