# Supplementary material for: Stabilizing the Anode and Cathode Interface Synchronously via Electrolyte-Triggered Hydrogel Interphase for Zinc Metal Batteries
Source: Nanomicro Lett. 2026 Jan 13;18:206. doi: 10.1007/s40820-025-02051-1 (PMC12799898; doi:10.1007/s40820-025-02051-1)
Supplement: Supplementary file 1 — Supplementary file1 (DOCX 20679 kb) [file 40820_2025_2051_MOESM1_ESM.docx]

Supporting Information for

**Stabilizing the Anode and Cathode Interface Synchronously *via* Electrolyte-Triggered Hydrogel Interphase for Zinc Metal Batteries**

Xinze Cai^1^, Xin Li^1^, Jiahui Liang^1^, Jiazhen Qiu^1^, Wenkuo Lin^1^, Chunlong Dai^1^, Zifeng Lin^1,^ *, Jiangqi Zhao^1, 2,^ *

^1^College of Materials Science and Engineering, Sichuan University, Chengdu 610065, P. R. China

^2^State Key Laboratory of Polymer Materials Engineering, Polymer Research Institute at Sichuan University, Chengdu 610065, P. R. China

*Corresponding authors. E-mail: [linzifeng@scu.edu.cn](mailto:linzifeng@scu.edu.cn) (Zifeng Lin); [Jiangqizhao@scu.edu.cn](mailto:Jiangqizhao@scu.edu.cn) (Jiangqi Zhao)

**Supplementary Figures**


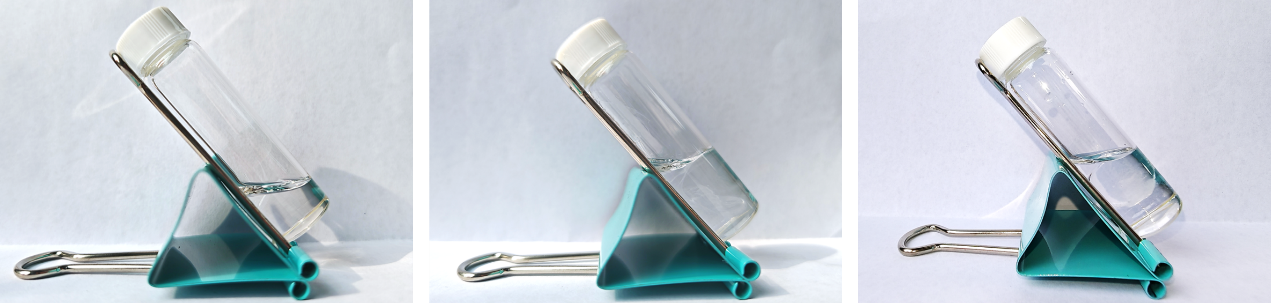


Filling 2 M ZnSO_4_ electrolyte

CPS

Electrolyte-triggered gelation

**Fig. S1** Optical photos of the solvent exchange gelation process

**Discussions on Fig. S1**

Initially, the CPS solution is homogeneous and transparent. Upon 2 M ZnSO_4_ electrolyte filling, the CPS solution undergoes a solvent exchange reaction, resulting in a change in the transmittance of visible light in partial regions. Following the completion of gelation, a transparent hydrogel manifests within the specimen.

**Fig. S2** Ramna spectra of 2 M ZnSO_4_ electrolyte before and after solvent exchange

**Discussions on Fig. S2**

Raman spectroscopy reveals that, compared to the original 2 M ZnSO_4_ electrolyte, the residual electrolyte after gelation (Ex - 2 M ZnSO_4_) exhibits characteristic peaks of DMAc at 748 cm^-1^ and 1000 cm^-1^, confirming the solvent exchange between DMAc and H_2_O. Furthermore, detailed analysis of the SO_4_^2-^ stretching vibration peak revealed higher contact ion pair (CIP) content between SO_4_^2-^ and Zn^2+^ in the exchanged electrolyte. This finding indicates that trace residual DMAc solvent alters the electrolyte properties, promoting SO_4_^2-^ enrichment within the Zn^2+^ solvation structure.

**Fig. S3** Finite element simulation of interfacial environment of the BZ anode. **a** Distribution of electric field and **b** concentration field

**Discussions on Fig. S3**

From a lateral perspective, the combination of the restricted contact between the electrode and the separator, and the incomplete wetting of the electrolyte, results in an ineffective interface region, which reduce the electrode utilization and exacerbate the disturbance of the interfacial environment.

**Fig. S4** Finite element simulation of interfacial environment of the anode with hydrogel interphase. **a** Distribution of electric field and **b** concentration field

**Discussions on Fig. S4**

This strategy, exploiting the highly fluid of the precursor solution facilitates the establishment of the seamless interface between the electrode and the separator, thereby homogenizing the distribution of the electric field and ionic concentration at the interface.

**Fig. S5** Finite element simulation of interfacial environment of different anodes. **a** Distribution of zinc concentration on the BZ and the hydrogel interphase modified anode **b** along the direction of SEI thickness

**Fig. S6** The statistic of current density distribution on different anodes

**Discussions on Fig. S6**

The statistical distribution of the interfacial current density demonstrates that enhancing the ion diffusion efficiency through the SEI can enable a more uniform redistribution of the interfacial electric field and exert long-term modulation from the initial stage to the final plating stage.

**Fig. S7** The Change of deposition reaction rate on different anodes during the plating process. **a** The BZ anode. **b** The hydrogel interphase modified Zn anode

**Discussions on Fig. S7**

The simulation results of the deposition rate imply that unavoidable minute protrusions on the anode surface result in a significant difference in the deposition rate of different areas of the anode at the initial deposition. Furthermore, the slow diffusion of ions within the SEI exacerbates this inhomogeneous deposition behavior, which in turn gradually induces the growth of dendrites. Enhancing the ion diffusion efficiency through the SEI can effectively reduce the difference in deposition rates in different areas, thereby avoiding zinc dendrites.

**Fig. S8** Temperature-dependent electrochemical impedance spectra of Zn symmetric cells with different anodes. **a** The BZ anode. **b** The hydrogel interphase modified Zn anode

**Fig. S9** Optical photographs of different anodes after cycling at a current density of 1 mA cm^-2^. **a** The BZ anode. Scale bar, 0.5 mm. **b** The hydrogel interphase modified Zn anode. Scale bar, 0.5 mm


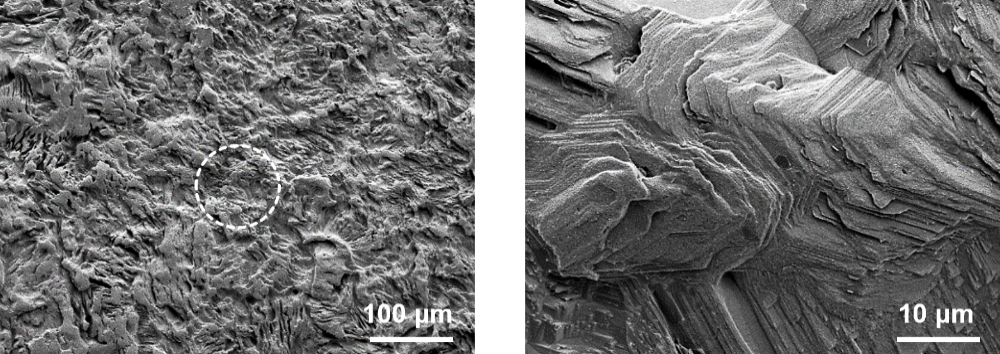


**Fig. S10** SEM images of hydrogel interphase modified anodes after critical current density test


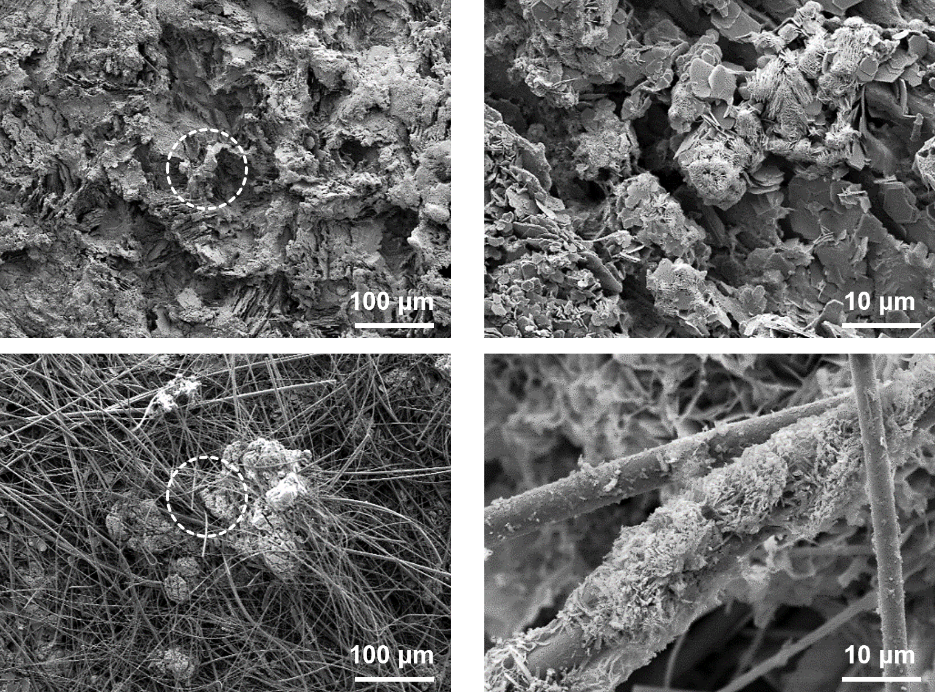


**Fig. S11** SEM images of BZ anodes after critical current density test

**Discussions on Fig. S11**

In addition to the dendritic deposits and irregular stripping pits, the co-deposits of glass-fiber and Zn further exacerbating the inhomogeneity of the deposition layer on the BZ surface.

**Fig. S12** The surface roughness of different anodes after critical current density test

**Fig. S13** Nyquist plots of the *in situ* EIS test with different anodes. **a** The BZ anode. **b** The hydrogel interphase modified Zn anode

**Fig. S14** The corresponding DRT results. **a** The BZ anode. **b** The hydrogel interphase modified Zn anode


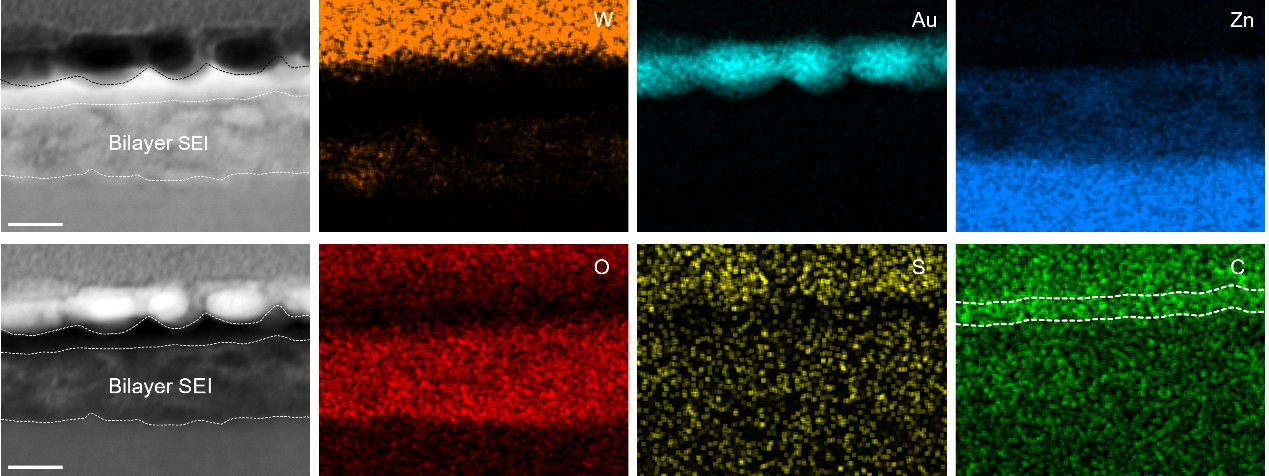


**Fig. S15** Bright-field TEM image, HAADF image and corresponding EDS mappings of the hydrogel interphase induced SEI. Scale bar, 10 nm

**Fig. S16** The elemental content variations of different anodes in the thickness direction after cycling. **a** The BZ anode. **b** The hydrogel interphase modified Zn anode

**Discussions on Fig. S16**

Fig. S16 demonstrates the content changes of C, O, N, S, and Zn elements as the sputtering time increases. In the unetched state, both anode surfaces exhibit relatively high C content, which can be attributed to surface C contamination. Therefore, the SEI composition analysis for both anodes is conducted after 30 s of etching. As shown in Fig. S16b, the content of C and N in the hydrogel interphase induced SEI underwent a significant decrease in proportion with the increase in etching time, while the content of S reached a peak after 120 s of etching and then underwent a slight decrease. This provides strong evidence for the bilayer structure of the hydrogel interphase induced SEI. It is noteworthy that after 360 s of etching, the Zn content within the hydrogel interphase induced SEI surpassed the O content, suggesting the near-complete etching of the SEI and consequent exposure of pristine metal surfaces on the anode. However, under the same etching duration, the O content within the formative SEI consistently exceeds that of other elements, indicating the greater thickness of the formative SEI (Fig. S16a).

**Fig. S17** XPS spectra with depth profiles of N 1*s* for the Zn anode with hydrogel interphase after cycling

**Fig. S18** Zn LMM Auger spectra of the Zn anode with hydrogel interphase

**Discussions on Fig. S18**

The Zn LMM Auger spectra is useful in determine the Zn chemical states. The peak at 988 eV is the ZnO Auger line. With increasing sputtering time, the ZnS Auger line (989 eV) and the Zn Auger line (994.7 eV) consequently appear. When combined with the Zn 2*p* spectra, it can be deduced that the hydrogel induced SEI is primarily composed of ZnS and a minor amount of ZnO.

**Fig. S19** XPS spectra with depth profiles of Zn 2*p* for the Zn anode with hydrogel interphase after cycling

**Fig. S20** TOF-SIMS 3D and 2D distributions of CNO^-^, C^-^, Zn_2_O^-^ and ZnSO_4_^-^ from the hydrogel interphase induced SEI

**
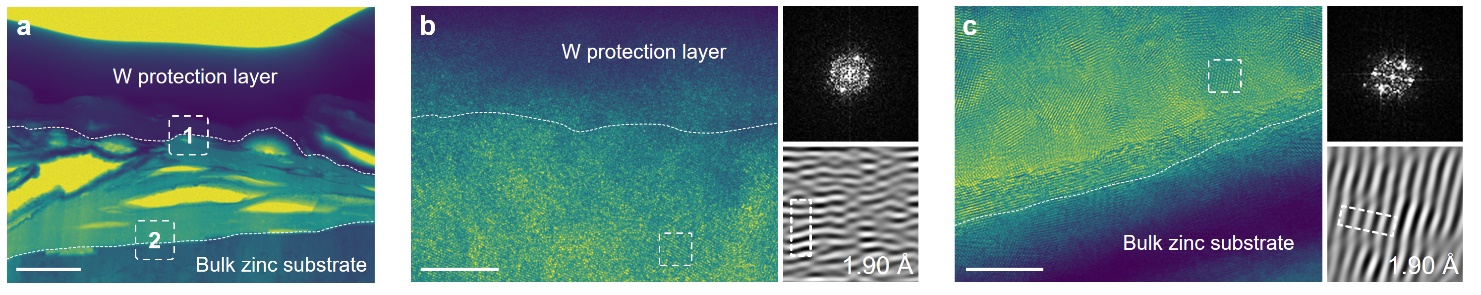
**

**Fig. S21** Structural and component characterizations of the formative SEI on the BZ anode. **a** SEM images of BZ anodes cycled for 20 cycles. Scale bar, 500 mm. **b** and **c** HRTEM images, the fast Fourier transform images and corresponding inverse fast Fourier transform images collected at specific regions of BZ anodes cycled for 20 cycles. Scale bar, 5 nm

**Discussions on Fig. S21**

FIB prepared cross-section of the BZ anode following cycling reveals that the formative SEI exhibits characteristics of substantial thickness, inhomogeneity, and high porosity, with an average thickness of 800 nm. High-resolution transmission electron microscopy (HRTEM) further reveals that the formative SEI consists of a large amount of low crystallinity zinc sulfate hydroxide hydrate.

**
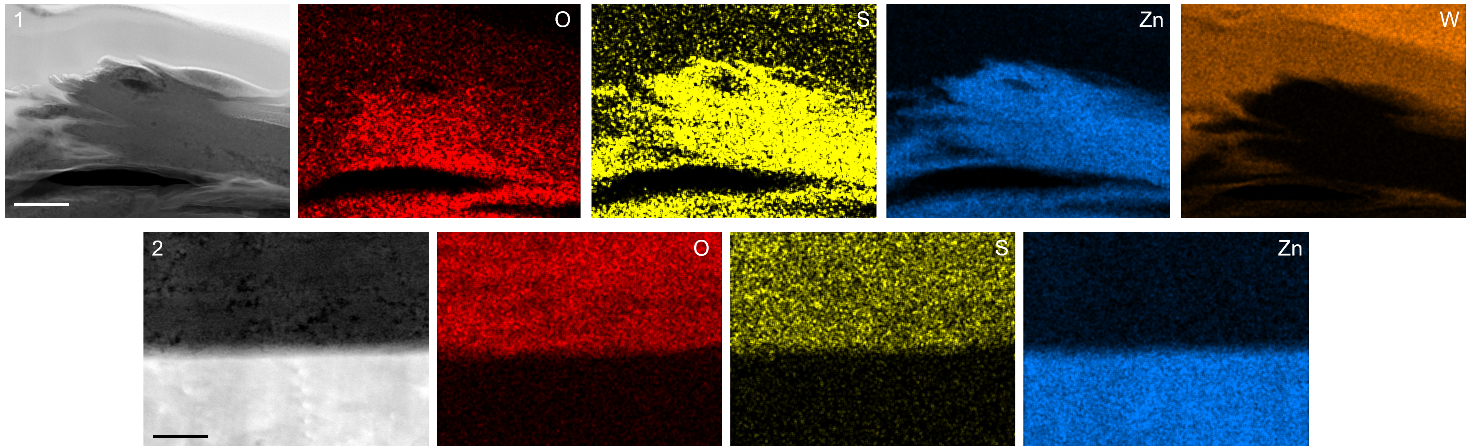
**

**Fig. S22** HAADF image and corresponding EDS mappings of the hydrogel interphase induced SEI. Scale bar, 100 nm

**Fig. S23** XPS spectra with depth profiles of C 1*s* and N 1*s* for the BZ anode after cycling

**Discussions on Fig. S23**

The C 1*s* signal of the BZ anode demonstrates two peaks at 284.8 and 293.9 eV, assigned to C-C bond and carbonate, which are gradually eliminated upon depth profiling. The N 1*s* signal of the BZ anode is also negligible, indicating a minimal organic presence in the formative SEI on the BZ anode, with only a negligible amount of contaminated carbon yielding the C 1*s* signal.

**Fig. S24** XPS spectra with depth profiles of Zn 2*p* for the BZ anode after cycling

**Discussions on Fig. S24**

Due to the negligible binding energy shift of Zn in the Zn 2*p* region, the peaks observed at 1046.8 eV and 1023.6 eV in the Zn 2*p* spectra are tentatively attributed to the Zn-O or Zn-O-H bonds.

**Fig. S25** Zn LMM Auger spectra of the BZ anode

**Discussions on Fig. S25**

The Zn LMM Auger spectra of the BZ anode reveals a peak at 986.2 eV, ascribed to Zn(OH)_2_ Auger line. This peak shifts to 988 eV, which is assigned to the ZnO Auger line, as the sputtering time increases. Furthermore, combining Zn *2p* spectra allows the conclusion to be drawn that the surface layer of the formative SEI on the BZ anode is mostly zinc sulfate hydroxide hydrate, while the interior contains ZnO.


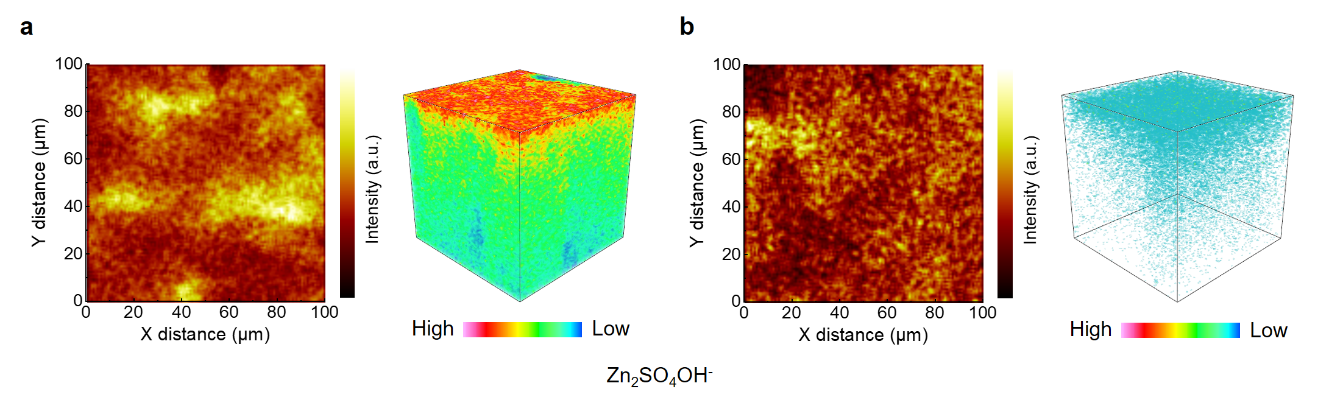


**Fig. S26** TOF-SIMS 3D/2D distributions of Zn_2_SO_4_OH^-^ from SEIs formed on different anodes. **a** The BZ anode. **b** The hydrogel interphase modified Zn anode

**Discussions on Fig. S26**

The vertical distribution of Zn_2_SO_4_OH^-^ fragments demonstrates a rapid decrease in signal intensity along the interphase thickness, with an overall negligible intensity, indicating the hydrogel interphase induced SEI contains minimal alkali sulphate component. Conversely, the formative SEI on the BZ anode contains substantial quantities of alkali sulfate components throughout the etching region. These components are noticeably aggregated at the surface, which is consistent with the results of the XPS tests.

**Fig. S27** XRD curves of anodes after cycling

**Discussions on Fig. S27**

The physical phases of the main components of SEIs on Zn anodes were further determined by X-ray diffraction (XRD). The analytical results showed that the formative SEI on the BZ anode is primarily composed of zinc sulfate hydroxide hydrate, whilst the hydrogel interphase induced SEI almost does not contain this component.

**Fig. S28** XPS spectra with depth profiles of S 2*p* for the BZ anode after cycling

**Fig. S29** TOF-SIMS 3D or 2D distributions of Zn_2_O^-^ and ZnSO_4_^-^ from the BZ anode


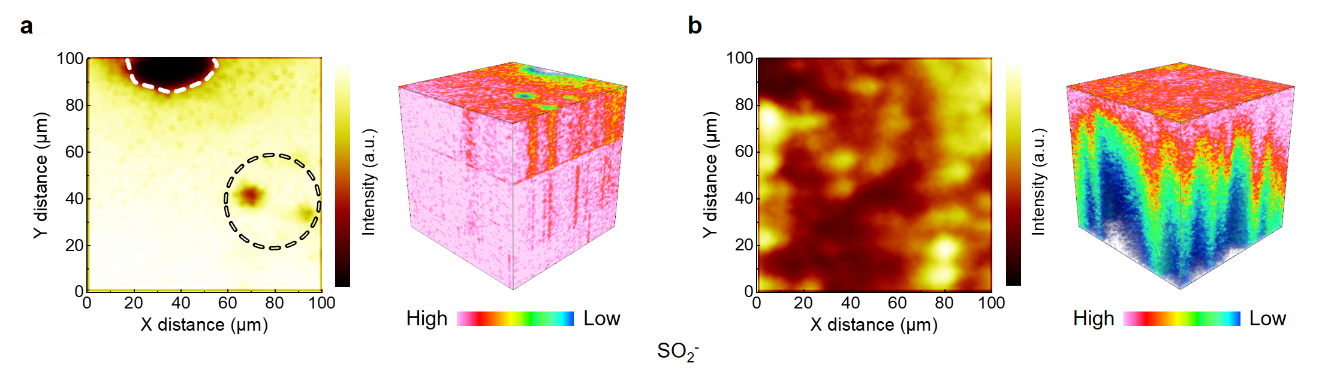


**Fig. S30** TOF-SIMS 3D/2D distributions of SO_2_^-^ from SEIs formed on different anodes. **a** The BZ anode. **b** The hydrogel interphase modified Zn anode

**Discussions on Fig. S30**

In comparison with the signal intensities of Zn_2_SO_4_OH^-^, those of Zn_2_O^-^ and ZnSO_4_^-^ fragments are significantly lower and unevenly distributed along the thickness of the interphase, with the overall higher signal intensities in the inner layer than in the outer layer, indicating the inner part of the formative SEI on the anode of the BZ contains a small amount of anion-derived SEI component due to the decomposition of SO_4_^2-^. Nevertheless, alkali sulphate remains dominant.

**Fig. S31** Normalized intensity profiles of different fragments. **a** BZ anode. **B** hydrogel interphase modified Zn anode

**Discussions on Fig. S31**

By normalizing the intensity of each fragment relative to its maximum value, the normalized intensity profile of the several fragments of interest is plotted. As demonstrated in Fig. S31b, for the hydrogel interphase induced SEI, the normalized intensity of organic component characteristic fragments (C_2_HO^-^ and CNO^-^) attained its peak earlier than that of inorganic component characteristic fragments (ZnSO^-^ and Zn_2_O^-^), further corroborating the structural characteristics of the organic outer layer and inorganic inner layer in the bilayer SEI. For the formative SEI on the bare zinc anode, the normalized intensity of characteristic fragments representing the alkaline salt passivation layer (Zn_2_SO_4_OH^-^) gradually decreases as sputter time increases, while remaining at a consistently high level (Fig. S31a). In contrast, the normalized signal intensity of characteristic fragments representing anion-derived inorganic components (ZnSO^-^ and Zn_2_O^-^) continuously increases with sputter time. The results suggests that the formative SEI is predominantly composed of electrochemically inert alkaline sulfates, and anion-derived inorganic components exist only internally, further confirming the thermodynamic instability of the formative SEI in aqueous electrolyte systems.

**Fig. S32** The nanoindentation test of different anodes after cycling

**Fig. S33** Mechanical properties of SEIs formed on different anodes

**Fig. S34** The LSV curves of different electrodes in the 2M ZnSO_4_ electrolyte

**Fig. S35** Phase analysis of the cathode material. **a** The SEM image of the MnO_2_ particles. Scale bar, 500 nm. **b** The XRD curves of the MnO_2_ particles

**Discussions on Fig. S35**

SEM and X-ray diffraction (XRD) were conducted to determine the phase and morphology of the synthesized MnO_2_ active materials. The outcomes of this analytical investigation indicate that the synthesized MnO_2_ adopts the alpha phase and exhibits a nanorod configuration.


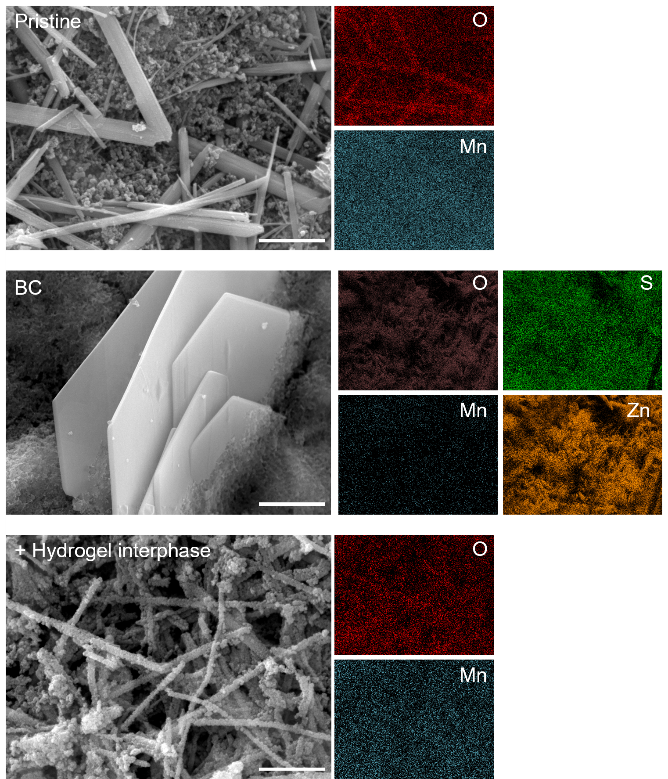


**Fig. S36** SEM images of different cathode. Scale bar, 1 µm

**Fig. S37** SEM images of Zn anodes in Zn/MnO_2_ full cells after 2000 cycles. **a** The BZ anode. **b** The hydrogel interphase modified Zn anode

**Discussions on Fig. S37**

The morphology of cycled anodes was analyzed by SEM. As illustrated in Fig. S37a, the surface of the cycled BZ exhibits both extremely deep stripping pits and a large amount of accumulated dendritic deposits. This severe asymmetric plating/stripping behavior accelerates the anode failure, leading to rapid capacity degradation of the full cell. Conversely, the surface of the hydrogel interphase modified Zn anode remains flat after cycling, and the deposited zinc flakes are densely packed, further demonstrating the important role of the hydrogel interphase construction on the stability of the Zn anode (Fig. S37b).

**Fig. S38** The discharging capacity and capacity retention rate (compared to the initial capacity at 0.5 C) of Zn/MnO_2_ full cells with different charging/discharging rates

**Fig. S39** Contour plots of CV tests after different numbers of cycles. **a** CV test of Zn/MnO_2_ full cells without and **b** with hydrogel interphase

**Fig. S40** Nyquist plots of EIS tests after different numbers of cycles. **a** EIS test of Zn/MnO_2_ full cells without and **b** with hydrogel interphase
